# Mechanistic Issues of the Interaction of the Hairpin-Forming Domain of tBid with Mitochondrial Cardiolipin

**DOI:** 10.1371/journal.pone.0009342

**Published:** 2010-02-22

**Authors:** François Gonzalvez, Fabrizio Pariselli, Olivier Jalmar, Pauline Dupaigne, Franck Sureau, Marc Dellinger, Eric A. Hendrickson, Sophie Bernard, Patrice X. Petit

**Affiliations:** 1 Institut Cochin, CNRS UMR8104 (Centre National de la Recherche Scientifique), INSERM U 567, Université Paris-Descartes, Paris, France; 2 Université Paris Descartes, UPR2228 CNRS (Centre National de la Recherche Scientifique), Paris, France; 3 BioMoCeti, CNRS UMR7033 (Centre National de la Recherche Scientifique), Université Paris VI - Université Paris XIII, GENOPOLE Campus 1, Site SERONO, RN 7, Evry, France; 4 USM 504 Biologie Fonctionnelle des Protozoaires, Muséum National d'Histoire Naturelle, Paris, France; 5 Department of Biochemistry, Molecular Biology, and Biophysics, University of Minnesota Medical School, Minneapolis, Minnesota, United States of America; 6 UPR 2228 CNRS (Centre National de la Recherche Scientifique), Université Paris Descartes, IFR 95, Paris, France; Baylor College of Medicine, United States of America

## Abstract

**Background:**

The pro-apoptotic effector Bid induces mitochondrial apoptosis in synergy with Bax and Bak. In response to death receptors activation, Bid is cleaved by caspase-8 into its active form, tBid (truncated Bid), which then translocates to the mitochondria to trigger cytochrome c release and subsequent apoptosis. Accumulating evidence now indicate that the binding of tBid initiates an ordered sequences of events that prime mitochondria from the action of Bax and Bak: (1) tBid interacts with mitochondria via a specific binding to cardiolipin (CL) and immediately disturbs mitochondrial structure and function idependently of its BH3 domain; (2) Then, tBid activates through its BH3 domain Bax and/or Bak and induces their subsequent oligomerization in mitochondrial membranes. To date, the underlying mechanism responsible for targeting tBid to mitochondria and disrupting mitochondrial bioenergetics has yet be elucidated.

**Principal Findings:**

The present study investigates the mechanism by which tBid interacts with mitochondria issued from mouse hepatocytes and perturbs mitochondrial function. We show here that the helix αH6 is responsible for targeting tBid to mitochondrial CL and disrupting mitochondrial bioenergetics. In particular, αH6 interacts with mitochondria through electrostatic interactions involving the lysines 157 and 158 and induces an inhibition of state-3 respiration and an uncoupling of state-4 respiration. These changes may represent a key event that primes mitochondria for the action of Bax and Bak. In addition, we also demonstrate that tBid required its helix αH6 to efficiently induce cytochrome *c* release and apoptosis.

**Conclusions:**

Our findings provide new insights into the mechanism of action of tBid, and particularly emphasize the importance of the interaction of the helix αH6 with CL for both mitochondrial targeting and pro-apoptotic activity of tBid. These support the notion that tBid acts as a bifunctional molecule: first, it binds to mitochondrial CL via its helix αH6 and destabilizes mitochondrial structure and function, and then it promotes through its BH3 domain the activation and oligomerization of Bax and/or Bak, leading to cytochrome c release and execution of apoptosis. Our findings also imply an active role of the membrane in modulating the interactions between Bcl-2 proteins that has so far been underestimated.

## Introduction

Over the past 15 years, it has become unequivocally clear that mitochondria play a crucial in the execution of apoptosis (Kroemer et al. 1995; Wang 2001). Most pro-apoptotic stimuli require a mitochondria-amplificatory step, which involves the disruption of the mitochondrial membrane potential (Vayssière et al. 1994; Petit et al. 1995; Zamzami et al. 1995b; Zamzami et al. 1995a) and the permeabilization of the mitochondrial outer membrane (MOMP) to apoptogenic factors, such as cytochrome c and Smac. Release of these factors into the cytosol triggers different cellular programs that culminate with the activation of caspases, committing cells to death. MOMP is tightly regulated by the proteins of the Bcl-2 family, which are divided into three subfamilies according to their function and homology shared within four Bcl-2-homology domains (designated BH1-4). The anti-apoptotic members (e.g., Bcl-2 and Bcl-XL) contain all four BH domains and repress the release of apoptogenic factors from mitochondria. In contrast, pro-apoptotic members promote MOMP and are subdivided into two groups: the multi-domains proteins (e.g., Bax and Bak) containing the 3 domains BH1-3 and the BH3 only proteins (e.g., Bid, Bad and Bim) [1]. Bcl-2 and Bcl-X_L_ are mitochondrial integral proteins and present a hydrophobic groove on their surface that can bind to the BH3 domain of the pro-apoptotic members, thereby neutralizing their action [2], [3]. The ability of the Bcl-2 proteins to hetero-dimerize among themselves provides the basis for the “life/death” rheostat [4]. In response to death signals, Bax and Bak are activated, directly and/or indirectly [5], [6], by the BH3-only proteins and undergo conformational changes that result in their stable insertion and subsequent oligomerization in the mitochondrial outer membrane [7], [8]. The BH3 only protein Bid relays the apoptotic signal from the extrinsic death receptors to the mitochondria in type II cells [9]. Following death receptor activation, Bid is cleaved by caspase-8 into its active form tBid, which rapidly translocates from the cytosol to the mitochondria and triggers cytochrome *c* release [10], [11]. Importantly, tBid interacts with mitochondria at the contact sites between the inner and outer membranes via a specific binding to cardiolipin (CL) [12], [13]. The pro-apoptotic activity of tBid was first thought to be solely limited to its BH3 domain by activating Bax and Bak. However, accumulating evidences strongly indicate that tBid can signal apoptosis through BH3-independent mechanisms. tBid interacts with CL and induces a remodeling of the mitochondrial cristae independently of its BH3 domain [14], [15], [16]. Furthermore, we previously shown that binding of tBid to CL immediately inhibit ADP-stimulated respiration and phosphorylation rate independently of Bax and Bak (Gonzalvez et al. 2005a; Gonzalvez et al. 2005b). Notably, both cristae remodelling and perturbations of mitochondrial homeostasis occured in a similar time scale, suggesting that tBid affects mitochondrial inner membrane in a BH3-independent manner [13], [14]. In line with this, we showed that tBid interacts specifically interacts with CL monolayers and stabilizes them into microdomains [13]. Interestingly, CL was recently shown to also provide an anchor and an activating platform for caspase-8 on mitochondria essential for efficient Fas signalling in type II cells [17]. Thus, the localized activity of caspase-8 may ensure that the cleavage of Bid is accomplished where it is needed, on the surface of mitochondria.

The structures of Bcl-XL (Muchmore et al. 1996), Bcl-2 (Petros et al. 2001), Bax (Suzuki et al. 2000) and Bid (Chou et al. 1999; McDonnell et al. 1999) have been solved. Despite having opposing roles, these proteins present a similar three dimensional structure consisting of 6 to 8 amphipathic alpha helices folded into a globular domain [2]. However, in contrast to Bcl-2, Bcl-XL and Bax, Bid does not contain a hydrophobic transmembrane domain at its c-terminus [18]. Bid consists of eight α-helices (designated αH1 through αH8, respectively), of which αH3 contains the BH3 domain [19], [20]. Helices αH6 and αH7 are hydrophobic and form an anti-parallel hairpin structure that is surrounded by the six other amphipathic α-helices. Although the underlying mechanism responsible for the translocation of tBid to mitochondria has been investigated in the past, conflicting results have emerged and some clarification is still required. On one hand, targeting of tBid to mitochondria was shown to rely on post-translational myristoylation modifications at the N-terminal of tBid [21]. In contrast, other studies indicated that tBid does not required its N-terminal myristoylation sites to bind to mitochondria, but relies on a CL binding domain, consisting of either helices αH4, αH5 and αH6 [12] or αH6, αH7 and αH8 [22], [23], [24].

In this study, we show that the helix αH6 is responsible for targeting tBid to mitochondrial CL and disrupting mitochondrial bioenergetics. In particular, αH6 interacts with mitochondria through electrostatic interactions involving the lysines 157 and 158 and immediately induces an inhibition of state-3 respiration and a slight uncoupling of state-4 respiration. These changes may represent a key event that primes mitochondria for the action of Bax and Bak. In addition, we also demonstrate that tBid required its helix αH6 to efficiently induce cytochrome c release and apoptosis. Altogether, these results strongly support the importance of the interaction of the helix αH6 with CL for both mitochondrial targeting and pro-apoptotic activity of tBid.

## Results

### αH6 Is Necessary for Targeting tBid to the Mitochondria

**Figure 1:**
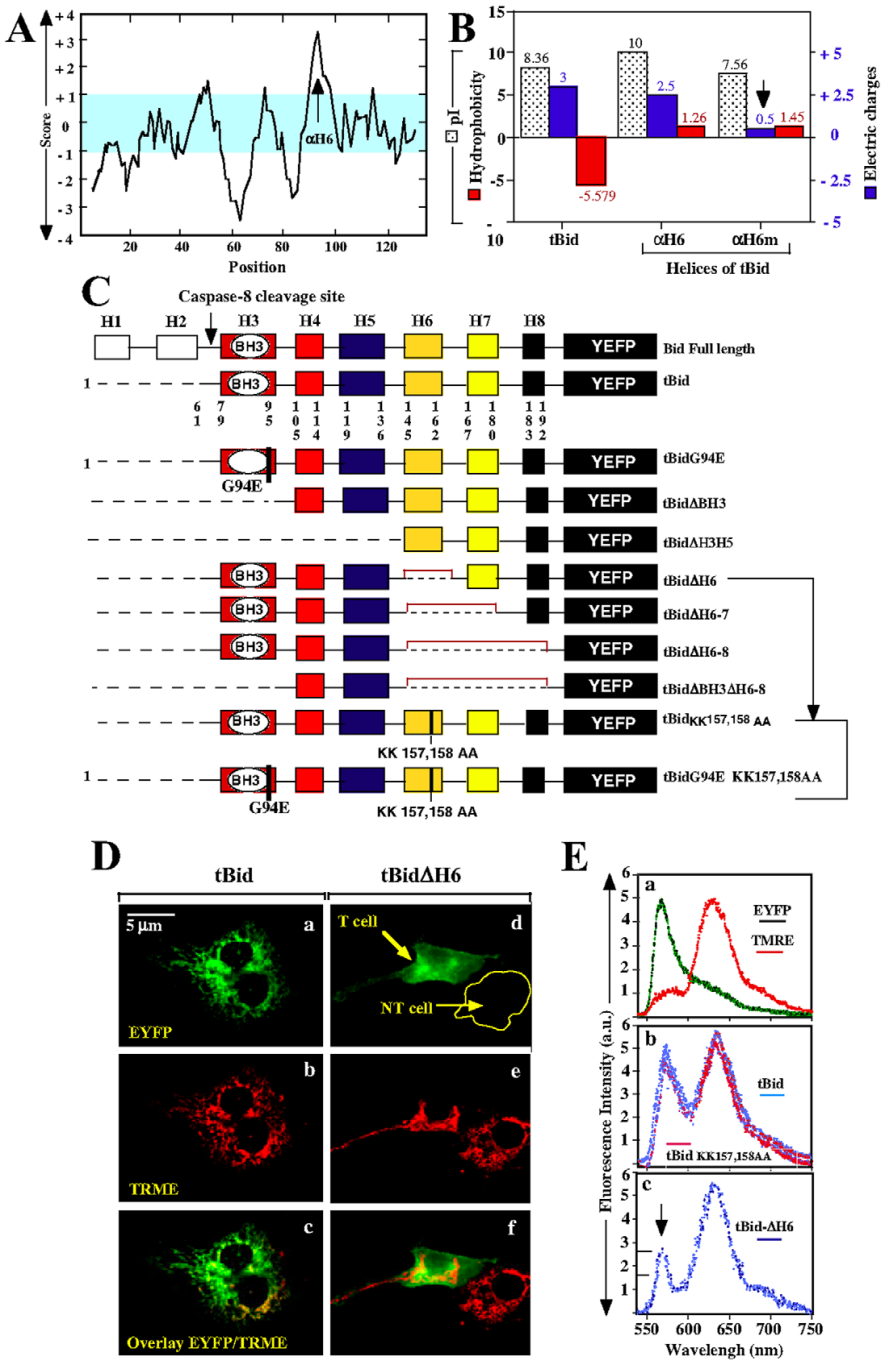
The helix αH6 targets tBid to the mitochondria. (A) and (B) Computer-based analysis of the biophysical properties of Bid. (A) Kite & Doolittle profile of Bid (PROSTCALE, Swiss Institute of Bioinformatics). (B) Comparison of the isoelectric points (pI), hydrophobicities and charges of tBid, αH6 and αH6m. (C) Schematic representation of tBid-EYFP mutants. (D) and (E) CV-1 cells were transfected with plasmids endoding tBid-EYFP mutants in presence of 10 µM of Bok-D to inhibit caspases activation and subsequent cell death. 24 h later, cells were stained with 20 nM of the mitochondrial potential probe TMRE and the localization of the tBid-EYFP mutants was determined using confocal microscopy (D) and microspectrofluorometry analysis (E).

In order to identify the domain responsible for targeting tBid to mitochondria, we performed a computer-based analysis of the biophysical properties of tBid (**Figure 1A, 1B** **and** **Table 1**). The Kyte-Doolittle hydropathy profile indicated that αH6 was the more amphiphilic helix of tBid with a hydophibicity of 1.265 and a net charge of 2.5, and *per se* the best candidate for a mitochondrial targeting domain (**Figure 1A and B** **,** **Table 1**).

**Table 1:** Biophysical properties of Bid and its alpha-helices: number of amino acids (AA), isolelectric point (pI), hydrophobicity and charge.

Names	AA number	pI	Hydrophobicity	Charge +/−
**Bid Full-length**	193	5,27	−0,488	−6,5
**Bid N-ter**	60	8,36	−0,579	+3
**H3**	21	5,57	−0,848	−0,5
**H4**	10	10,55	+0,930	+1
**H5**	18	3,90	−1,094	−3
***H6***	***18***	***10,50***	***+1,265***	***+2.5***
**H7**	14	7,55	−0,057	+0,5
**H8**	10	12,2	−1, 000	+3
***H6m***	***18***	***7,56***	***+1,456***	***+0,5***
**H6 + ½ H7**	27	9,99	+0,807	+3
**H6 + H7**	35	9,99	+0,650	+3
**H6 + H7 + H8**	47	10,93	+0,212	+5
**H4 + H5 + H6**	58	9,19	−0,340	+1,5
**BH3 Domain**	25	7,55	−0,596	−0,5

Other constructs like the hairpin forming domain αH6–H7 domain are described. The calculation of hydrophobicity was performed using the PROTSCALE software from the Swiss Institute of Bioinformatics according to the method of Kyte and Doolittle. αH6 and αH6m are presented in ***italic***.

Thus, we generated deletion mutants of tBid-EYFP (**Figure 1C**) and determined the ability of these mutants to translocate to the mitochondria by fluorescence microscopy and microspectroflurometry (**Figure 1D, 1E** **, and** **Table 2**). CV-1 cells transfected with tBid-EYFP construct show a punctate staining pattern that totally overlayed with the mitochondrial-specific stain TMRE (**Figure 1D** **, a to c**). Interestingly, deletion of αH6 completely abrogated the mitochondrial localization of tBid resulting in a diffuse cytosolic pattern throughout the cell (**Figure 1D** **, d to f**). These observations were confirmed by microspectrofluorometry (**Figure 1E** **and** **Table 2**). In CV-1 cells transfected with tBid-EYFP, EYFP and TMRE fluorescent peaks were of same intensity indicating that tBid was exclusively mitochondrial (Ratio = 1) (**Figure 1E** **, central panel**). Deletion of the alpha helices αH3, αH4, αH5, αH7 or αH8 did not affect the ratio between those two peaks, and thus localization of tBid to mitochondria (**Table 2**). In contrast, deletion of αH6 decreased the EYFP peak and led to a two folds decrease in peak ratio (0.53) (**Figure 1E** **, bottom panel**). These resullts strongly indicated that tBid depends on its helix αH6, and not on its BH3 domain, for its mitochondrial localization. In line with this, inactivation of the BH3 domain (tBidE94G) did not affect the ability of tBid to translocate to the mitochondria (**Table 2**). Moreover, while single deletion of αH7 and αH8 did not affect the targeting of tBid to mitochondria, deletion of those helices together with αH6 further decreased the peak ratio to 0.27 (**Table 2**), suggesting that αH6–8 may constitute the mitochondrial-binding domain of tBid. Consitent with this, αH6–8-GFP localized to mitochondria to the same extent as the full-length tBid-EYFP (**Table 2**).

**Table 2:** Intracellular localization of Bid-EYFP, tBid-EYFP and its deletion mutants.

Construct	EYFP/TRME	Localization
tBid	1±0,07	Mitochondrial
tBid_E_94_G_	1±0,07	Mitochondrial
tBidΔBH3	1±0,07	Mitochondrial
**tBidΔH6**	**0,53±0,05**	*Cytoplasmic*
**tBidΔH6–H7**	**0,30±0,03**	*Cytoplasmic*
**tBidΔH6–8**	**0,27±0,03**	*Cytoplasmic*
**tBidΔBH3ΔH6–8**	**0,27±0,03**	*Cytoplasmic*
***tBid_KK_157,158_AA_***	***0,94±0,40***	Mitochondrial*
***tBid_E_94_G,KK_157,158_AA_***	***0,94±0,54***	Mitochondrial*
tBidΔH3, or ΔH4, or ΔH5, or ΔH7, or ΔH8	0,97±0,09	Mitochondrial

CV-1 cells were transfected with plasmids encoding Bid-EYFP, tBid-EYFP or tBid-EYFP mutants in presence of 10 µM of Bok-D. 24 h later, cells were stained with 20 nM of the mitochondrial potential probe TMRE and the localization of the tBid-EYFP mutants was determined by calculation of the TMRE/EYFP ratios using microspectrofluorometry. A ratio of 1.0 indicates that EYFP exclusively colocalizes with TMRE at the mitochondria while a lower ratio indicates that EYFP is also localized to the cytosol. The * highlights the contructs that localize to the mitochondria but are unable to induce cytochrome *c* release or to alter mitochondrial bioenergetics. The constructs that localize to the cytoplasm are in *italic*.

### αH6 Recapitulates the Effect of tBid on Mitochondrial Bioenergetics

We previously showed that tBid inhibits ADP-stimulated respiration and phosphorylation rate independently of its BH3 domain [13]. Since αH6 was the only helix of tBid required for its mitochondria localization, we investigated whether αH6 recapitulates the effect of tBid on mitochondrial function. Purified αH6 was added on mice liver mitochondria and the basic bioenergetic properties of these mitochondria [oxidation rate (Voxidation) and mitochondrial membrane potential (ΔΨm)], were determined as described previously [13].

**Figure 2:**
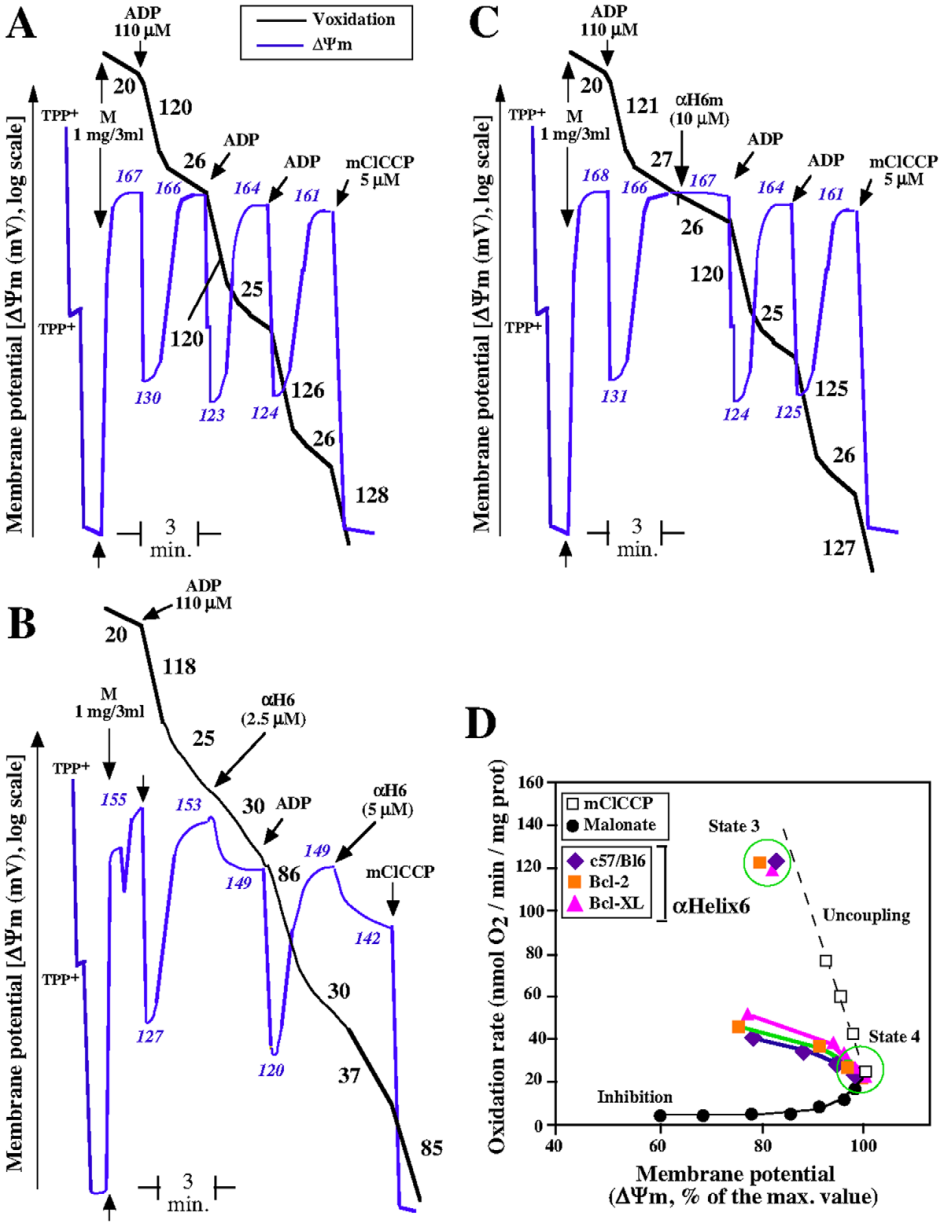
The helix αH6 affects mitochondrial bioenergetics. Purified mice liver mitochondria (M) were incubated in respiratory buffer (0.33 mg/ml). Oxygen consumption (Voxidation, black line) and mitochondrial potential (ΔΨm, bleu line) were monitored using a Clark-type electrode coupled to a tetraphenylphosphonium (TPP^+^) cation-sensitive electrode, as described previously [13]. 10 mM of succinate was added as oxidizable substrate (malonate is 1 mM). ADP was added to 110 µM and mCCCP was added to 10 µM. The numbers along the traces gives the values of oxidation rates in nmol O_2_/min/mg protein (Black) and mitochondrial potential in mV (Blue) (A) Oxygen consumption and potential of mitochondria oxidizing succinate. (B) and (C) Effect of the addition αH6 and αH6m on the oxygen consumption and mitochondrial potential. Each panels (A), (B) and (C) is representative of 3 independent experiments. (D) Representation of the effect of αH6 on oxidation rate and potential of mitochondria isolated from control, Bcl-2 and Bcl-X_L_ transgenic mice, as compared to uncoupling and inhibition of the mitochondrial respiration. Dotted black line shows the effect of the uncoupler CCCP, and the solid black line represents the inhibition of succinate-respiration by the specific inhibitor of complex II, malonate.

Like tBid [13], addition of aH6 on isolated mitochondria immediately increased state-4 respiration (to 120% of the initial state-4 respiration) and inhibited state-3 respiration (by approximately 25 to 30%) (Figure 2A and 2B). Inhibition of state-3 respiration was further amplified after a second addition of aH6 (5 mM). Importantly, aH6 also inhibited the uncoupling effect of mClCCP (carbonyl cyanide-m-chlorophenyl hydrazone) indicating that aH6 affected the electron transport chain (Figure 2B). As shown in Figure 2D, the effect of aH6 on mitochondrial potential and respiration is intermediate to those induced by the uncoupler mClCCP and the complex II inhibitor malonate. This suggests that aH6 induced bothuncoupling and inhibition of mitochondrial respiration. Importantly, aH6 had similar effects on mitochondria isolated from mice overexpressing Bcl-X_L_ and Bcl–2.

The findings that tBid specifically interacts with negatively charge mitochondrial CL [12], together with the observation that **α**H6 binds to mitochondria independently of its hydrophobicity [22] strongly suggested the involvement of electrostatic interaction. Notably, **α**H6 contains two highly conserved lysines at positions 157 and 158 [25]. Therefore, we asked whether these two lysines could be involved in electrostatic interactions with mitochondria. To test this, we synthesized a mutant form of **α**H6 (**α**H6m), in which the lysines 157 and 158 were replaced by two alanines (**Figure 1B**). This resulted in a loss of two positives charges but did not affect the hydrophobicity of the helix (**Table 1**). Mutation of these lysines completely abrogated the effect of the helix on mitochondrial bioenergetics, suggesting that **α**H6 required electrostatic interactions to interact with mitochondria (**Figure 2C**). However, mutation of the lysines 157 and 158 did not affect the mitochondrial localization of tBid (tBid_KK157158AA_, **Table 2**). This may account for the fact, that the helices αH7 and αH8 of tBid, which present a charge of +0.5 and +3 respectively, can engage electrostatic interactions with mitochondria (**Table 1**).

Altogether, these results show that αH6 recapitulates the effect of tBid on mitochondrial bioenergetics: αH6 interacts with mitochondria through electrostatics interactions and inhibits oxidative phosphorylation.

### αH6 Inserts into CL Monoloyer through Electrostatic Interactions and Destabilizes Them into Microdomains

We previously shown that tBid interacts specifically with artificial CL monolayers and stabilizes them into microdomains [17]. Using the same biophysical approach, we analyzed the ability of αH6 to interact with two differents CLs molecular species: (a) a natural Bovine Heart CL (BHCL), which contains mainly unsaturated acyl chains of linoleic acid (C18∶2), and (b) Tetra-Myristoylated CL (TMCL), a synthetic molecule which carries saturated myristoic acids (C14∶0).

**Figure 3:**
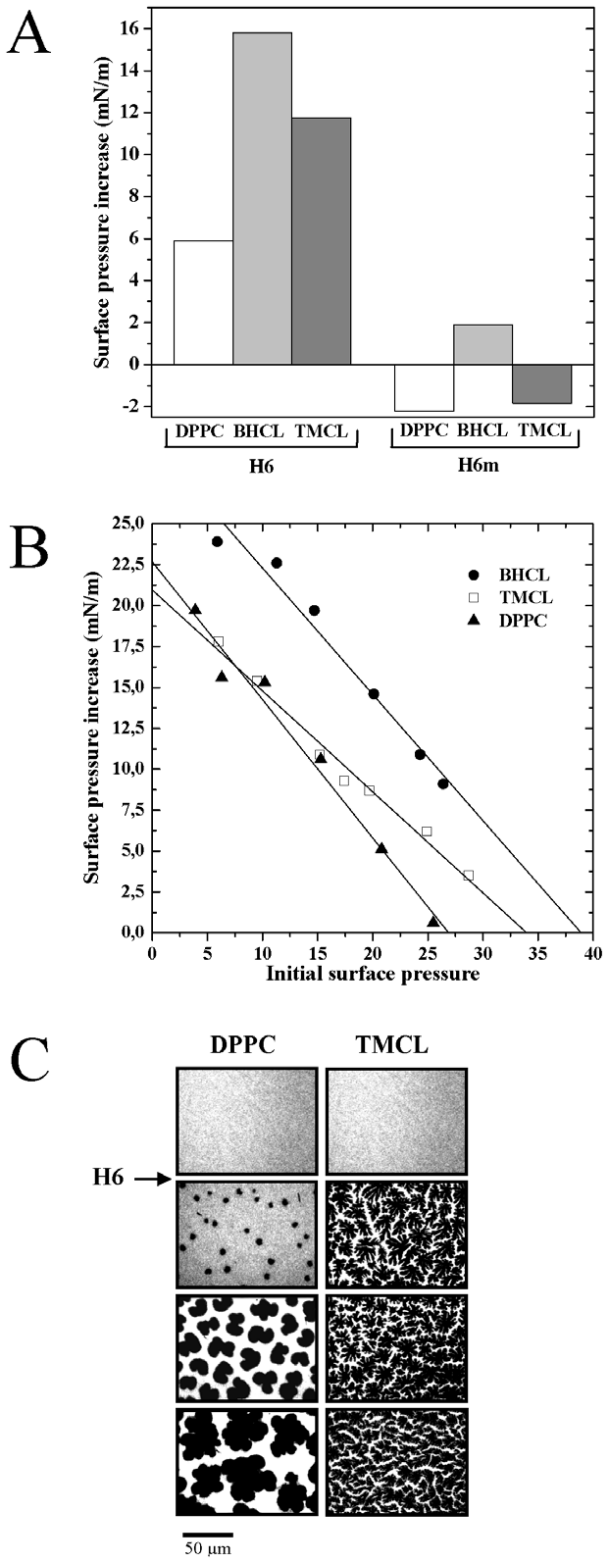
The helix αH6 specifically inserts into CL-monolayers through electrostatic interactions and reorganizes them into microdomains. Dipalmitoylphosphatidylcholine (DPPC), bovine heart CL (BHCL) and tetramyristoyl CL (TMCL) were spread at an initial surface pressure of 20 mN/m. αH6 and αH6m (1 µM) were injected into the subphase of these lipids monolayers. (A) The surface pressure changes (B) and epifluorescence images (C) were recorded as described previously (Gonzalvez et al 2005, CDD).

Monolayers of zwitterionic DPPC (dipalmitoylphosphatidylcholine) and negatively charged cardiolipin (BHCL and TMCL) were spread at an initial surface pressure of 20 mN/m. Addition of αH6 into the lipid subphase significantly increased their surface pressure (Δπ), indicating that αH6 inserted into those lipid monolayers (Figure 3A). Of note, this insertion was twice more important in presence CL monolayers and was completely inhibited after mutation of the lysines 157 and 158.

We then asked whether the interaction of αH6 with CL affect the organization of the phospholipid monolayer using epifluorescence microscopy. Before injection of αH6 into the subphase, lipid monlayers were in liquid-expanded phase and showed a uniform bright fluorescence. Addition of αH6 promoted the transition of the lipid monolayer to the liquid-condensed phase, as indicated by the appearance of dark probe-excluded domains. These domains were more rapidly formed in CL than DPPC monolayer and αH6m did not affect the organization of the phospholipid monolayer (Data not shown). Therefore like tBid, αH6 inserts specifically into cardiolipin monolayer through electrostatic interactions and stabilizes them into microdomains.

### αH6 and BH3 Domains Are Both Required for tBid-Mediated Apoptosis

**Figure 4:**
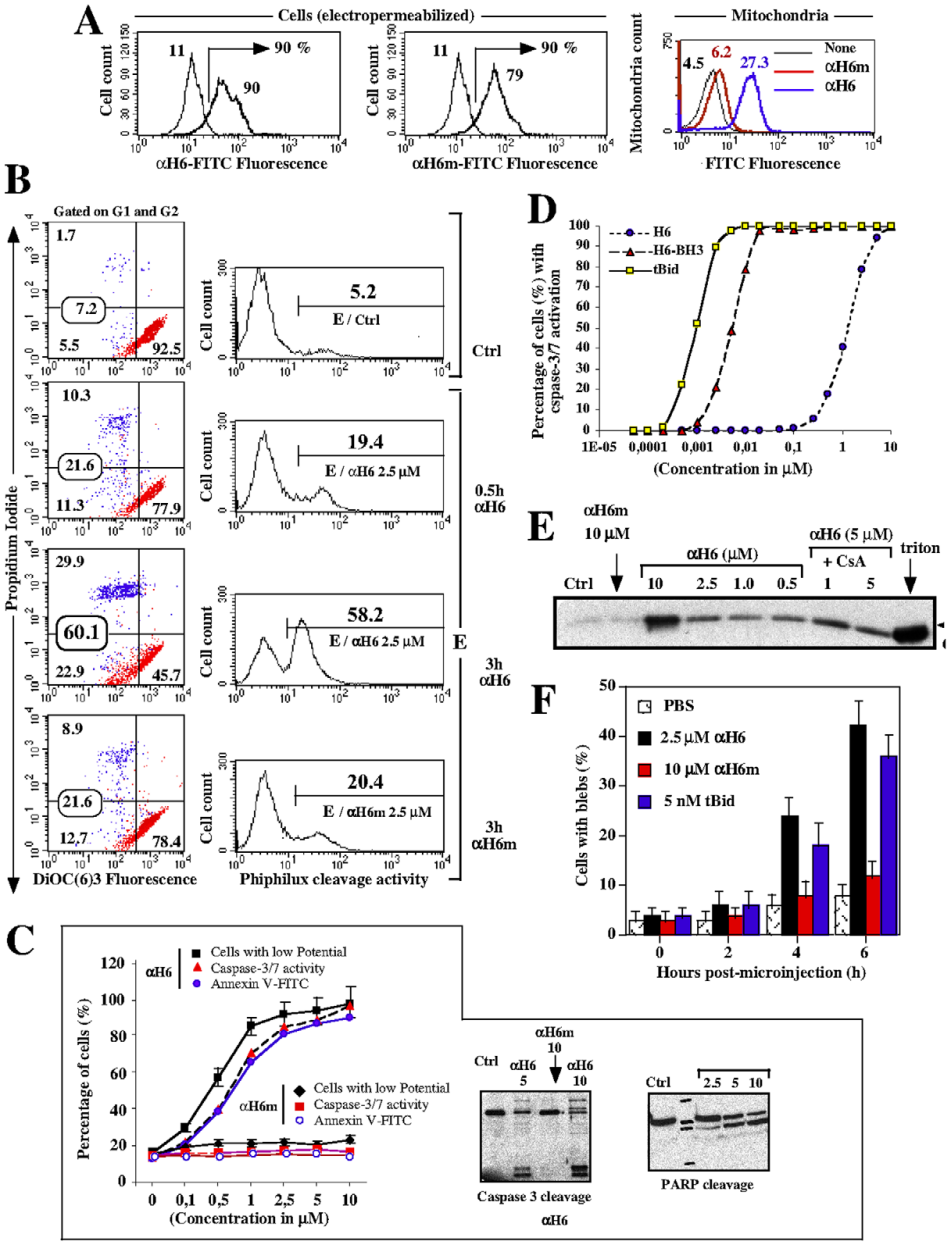
Electroporation and microinjection of αH6 induces apoptosis in Jurkat cells. Jurkat cells were electroporated with 2.5 µM of αH6-FITC and αH6m-FITC peptides, as described previously (Gabriel 2003). The efficiency of peptides electroporation into these cells was determined by measuring the FITC fluorescence using flow cytometry (A). Mitochondria were isolated from these cells, and analyze by flow cytometry described previously [36]. (B and C) Effect of αH6 and αH6m electroporation (2.5 µM) on mitochondrial potential, caspases 3/7 activation and cell viability in Jurkat cells. (B) Left panels: dot plot of DIOC(6)3 vs PI staining cells. Right panels: caspases 3/7 activities by cleavage of the Phiphilux substrate. E refers to electroporated cells. (C) Left pannels: dose response of αH6 and αH6m on mitochondrial potential, caspases 3/7 activation and apoptosis. Right panels: Caspase-3 and PARP cleavage were analyzed by Western Blotting. (D) Dose response curves of caspases 3/7 activation induced by electropermeabilization of tBid, αH6-BH3, and αH6 in Jurkat cells. (E) Jurkat cells were microinjected with tBid, αH6 and αH6m and the percentage of cell blebbing was measured by microscopy (F). Data are representative of 10 independant measurements (n = 10).

Since we showed that αH6 is required for targeting tBid to the mitochondria and recapitulates the effect of tBid on mitochondrial functions, we asked whether αH6 could also induce apoptosis. We electroporated αH6-FITC and αH6m-FITC peptides into J. Jhan cells [26] and analyzed their effect on mitochondrial membrane potential, caspase activation and cell viability by flow cytometry (**Figure 4**). Upon electropermeabilization, 90% of the viable cells incorporated αH6-FITC or αH6m-FITC (**Figure 4A** **, left and middle panel**). Consistent with our previous observations, only mitochondria isolated from cells electroporated with αH6-FITC exert a FITC-fluorescence (**Figure 4A, right panel**).

Electropermeabilization of αH6 induced a time and dose-dependent induction of apoptosis characterized by a drop of mitochondrial potential, activation of the effector caspase-3 and 7, PARP cleavage, externalization of phosphatidyl serine (**Figure 4B–D**). In contrast, electropermeabilization of **α**H6m did not lead to apoptosis (**Figure 4B–D**). To further confirm these results, we micro-injected αH6 and αH6m peptides into Jurkat cells (**Figure 4F**). As expected, micro-injection of 2.5 µM of αH6 induced blebbing of the cells after 4 hours, while αH6m had no effect. Notably, 2.5 µM of αH6 induced membrane blebbing with a similar kinetics as 5 nM of tBid.

By comparing the dose response curves of caspase 3/7 activation induced by electropermeabilized recombinant tBid and αH6 peptide (**Figure 4D**), it appeared that tBid is efficient at nM doses, while αH6 peptide showed an effect at µM range of concentrations. Interestingly, fusion of BH3 domain peptide to αH6 peptide strongly increased the activity of the peptide and shifts the effective dose to nM range. This suggested that both BH3 and αH6 domains may participate in the pro-apoptotic activity of tBid.

**Figure 5:**
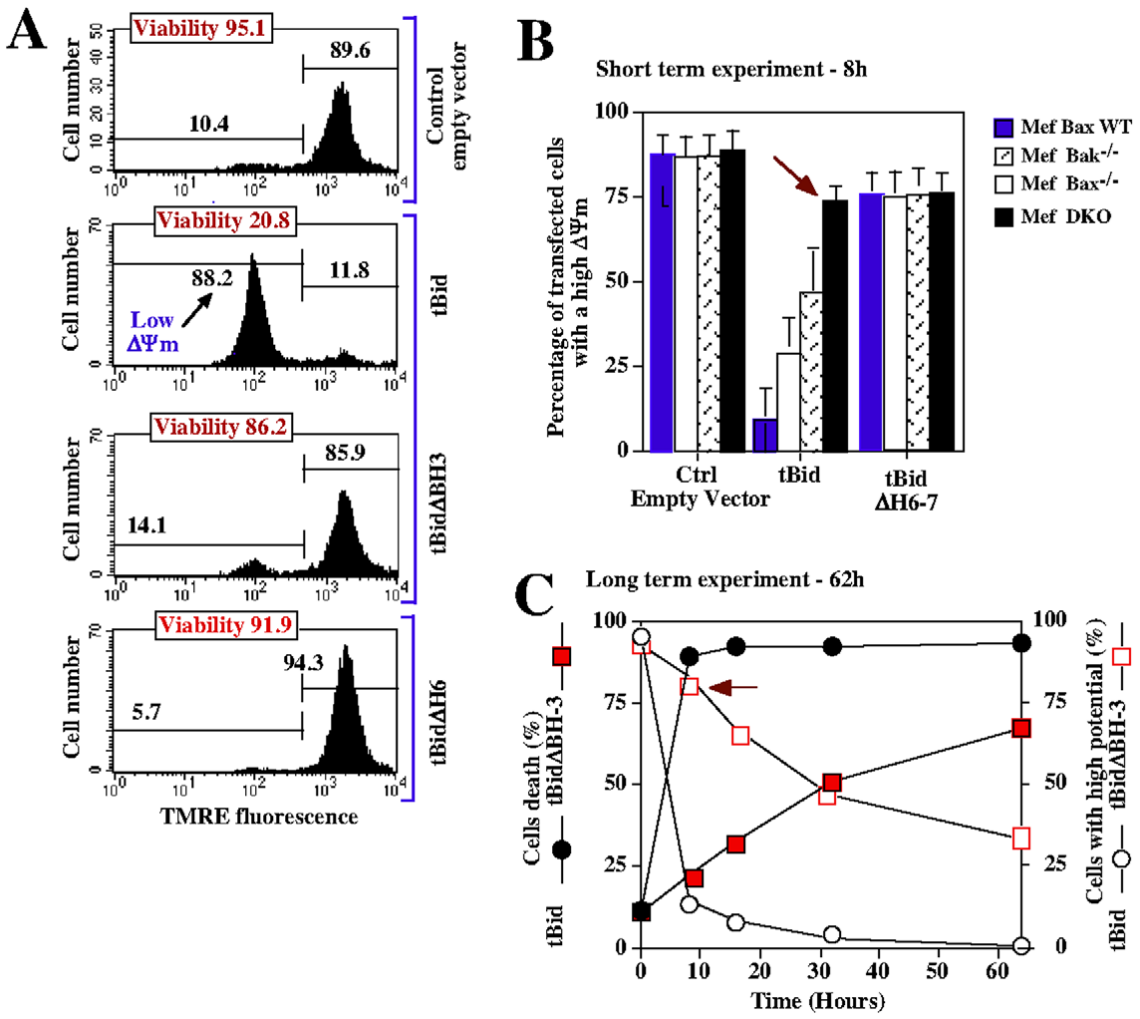
αH6 and BH3 domains are both required for tBid-induced apoptosis. (A) Jurkat cells were transfected with either empty vector control or with plasmids encoding tBid, tBidΔBH3 and tBidΔH6. 12 h latter mitochondrial membrane potential ΔΨm (TMRE) and cell viability (PI) were assessed by FACS analysis. Each panels is representative of 3 independent experiments. (B) Wild-type, Bax^+/+^, Bax^−/−^, Bak^−/−^ or double-knockout (DKO) Mefs cells were transfected with either empty vector control or with plasmids encoding tBid and tBidΔH6. After 8 h, green cells were analyzed by FACS for mitochondrial potential using TMRE. The red arrow indicates the ΔΨm in DKO Mefs. (C) Mefs cells were transfected with plasmids encoding tBid and tBidΔBH3, and the kinetics of mitochondrial depolarization and cell death were measured over 60 h by FACS analysis.

To determine the importance of both BH3 and αH6 domains for tBid-induced apoptosis, we compared the pro-apoptotic activity of tBid-EYFP constructs in HCT116 cells by monitoring mitochondrial membrane potential and cell viability (**Figure 5A**). While full length tBid-EYFP induced mitochondrial membrane depolarization in 88% of cells and lead to more than 80% of cell-death, single deletion of BH3 or αH6 domains strongly inhibited tBid-mediated apoptosis. Therefore, tBid relies on both BH3 and αH6 domains to induce apoptosis. We then took advantage of the Bax or Bak knock-out and double knock-out DKO MEFs cells to investigate the involvement of Bax and Bak in tBid-mediated apoptosis. Transfection of tBid in wild type, Bax-deficient or Bak-deficient MEFs incuced mitochondrial depolarization after 8 hours (**Figure 5B**). In contrast, DKO MEFs were strongly protected from tBid-induced apoptosis at this time point (red arrow) (**Figure 5B**). Consistent to our previous findings, tBidΔH6 did not induce apoptosis in MEFs. Importantly, tBid was able to induce mitochondrial potential depolarization and to kill DKO MEFs at latter time point, reaching 70% of cell death after 62 hours (**Figure 5C**). This may account for mitochondrial bioenergetic alteration and lipid peroxidation induced by the αH6 domains of tBid (data not shown). In line with this, deletion of αH6 completely protected the DKO MEFs from t-Bid mediated apoptosis whereas deletion of the BH3 domain had no effect.

**Figure 6:**
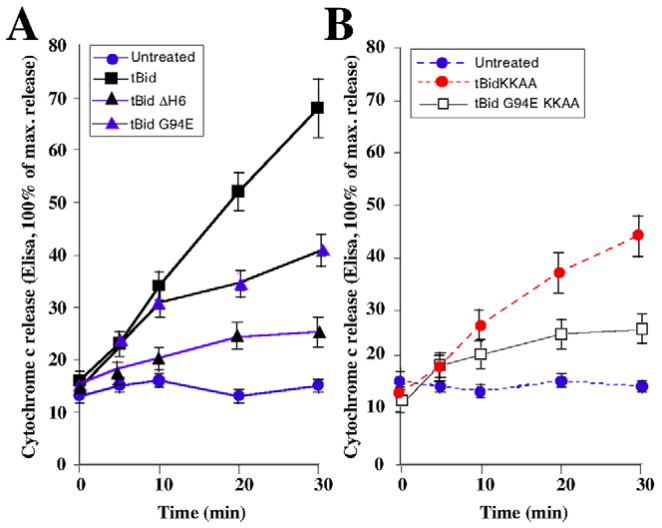
αH6 and BH3 domains are both required for tBid cytochrome *c* release activity. (A) and (B) Jurkat cells were electroporated with plasmids encoding tBid, tBidΔH6, tBidG94E, tBidKKAA and tBidG94EKKAA and the kinetics of cytochrome *c* release in the cytosolic fractions were detected by ELISA.

We previously showed that tBid is able to induce cytochrome *c* release via a BH3-independant mechanism involving transient opening of the PTP (permeability transition pore) [17]. Therefore, we next analyzed the role of the BH3 and αH6 domain in tBid-mediated cytochrome *c* release. Addition of recombiant tBid on isolated mitochondria resulted in a time-dependant release of cytochrome *c*, which reach 70% of the total cytochrome *c* after 30 minutes (**Figure 6**). As we expected from the lack of mitochondrial localization, deletion of αH6 strongly impaired the cytochrome *c* release activity of tBid (20% after 30 min). Importantly, inactivation of the BH3 domain (tBid G94E) or mutation of the two lysines 157 and 158 (tBid KKAA) had similar inhibitory effect on tBid-mediated cytochrome *c* release (40% after 30 min). Moreover, inactivation of the BH3 domain together with substitution of the two lysines (tBid_G94E,KKAA_) further inhibited the release of cytochrome *c* to the level tBidDH6 (20% after 30 min). This indicated that tBid induces cytochrome *c* release through BH3 dependant and BH3-independant mechanisms; the lastest involving electrostatic interactions through its αH6.

### tBid Relies on αH6, but Not on Its BH3 Domain, to Inducte Superoxide Anion Production and Mitochondrial Lipid Peroxidation

**Figure 7:**
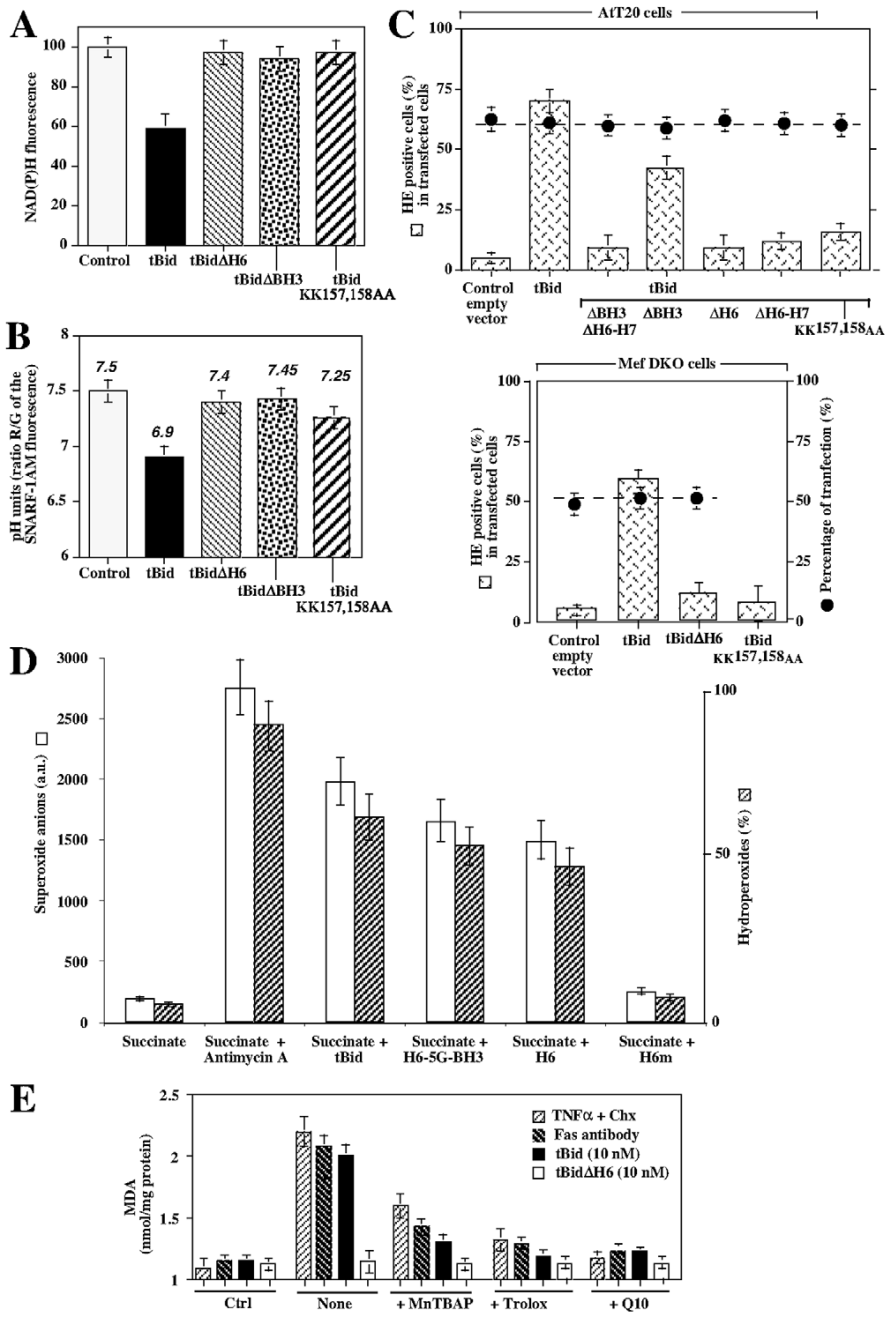
tBid required its helix αH6, but not its BH3 domain, to induce superoxide anion production and mitochondrial lipid peroxidation. (A) and (B) Wild-type hepatocytes were transfected with a control vector (control) or plasmids encoding tBid, tBidΔH6, tBidΔBH3 and tBidKKAA and NAD(P)H and SNARF-1AM fluorescence (pH indicator) were measured by FACS. Data are given as % of the control ± SD. pH units were determined using a calibration curve generated using nigericin-permeabilized cells kept in buffer of different pH values. (C) AtT20 cells or DKO Mefs were transfected with empty vector (control) or plasmids encoding tBid, tBidΔH6, tBidΔBH3, tBid tBidKKAA, tBidΔH6–H7, tBidΔBH3ΔH6H7. Cells were then stained with hydroethidine (HE, Invitrogen/Molecular probes) to measure superoxide anion production. The percentages of transfected cells are indicated by the dotted line. (D) Purified mice liver mitochondria were energized using succinate (+ rotenone) and treated using 10 nM tBid, H6-5G-BH4, αH6 and αH6m. Mitochondrial superoxide anion production was measured using HE (a.u.  =  arbitrary units) whereas hydroperoxide was measured using amplex red. (E) Wild-type hepatocytes were treated with TNFα/cycloheximide, anti-Fas antibody or transfected with a plasmids encoding tBid and tBidΔH6. Mitochondrial lipid peroxidation was measured by FACS using MDA. We used the antioxidants trolox (2 mM), MnTBAP (1 mM) and MitoQ10 (1 µM).

We have previously shown that tBid inhibits ADP-stimulated respiration and phosphorylation rate in isolated mitochondria through a BH3 independent mechanism in mice and yeast mitochondria [13]. Here, we investigated whether tBid affects cellular bioenergetics. Electroporation of tBid in hepatocytes lead to a decrease in cellular NADP(H) fluorescence associated with an acidification of the cytosol, while tBidΔH6 or tBid KK157,158AA had no effect (**Figure 7A and 7B**).

We examined whether tBid enhances superoxide anion production and lipid peroxidation in AtT20 cells. Transfection of tBid induced superoxide anion production in 60% of the cells (**Figure 7C** **, upper panel**). Deletion of αH6 or mutation of the lysines 157 and 158 completely abrogated the induction of superoxide anion in AtT20 cells. In contrast, deletion of the BH3 domain slightly decreased superoxide production. In line with this, tBid induced superoxide anion in Bax and Bak DKO MEFs cells (**Figure 7C** **, lower panel**). We also measured ROS (reactive oxygen species) production in purified mice liver mitochondria (**Figure 7D**). Addition of succinate to energize the mitochondria did not affect ROS level (superoxide anion and hydroperoxide) and inhibition of the electron transport chain using antimycin A strongly induced the production of ROS by mitochondria. Importantly, addition of tBid or the helix **α**H6 on isolated mitochondria increased ROS production in a similar manner. As expected, αH6m had no effect on mitochondrial ROS level.

Finally, we analyzed the effect of tBid and αH6 on mitochondrial lipid peroxidation in permeabilized-hepatocytes (**Figure 7E**). Addition of 10 nM of tBid induced mitochondrial lipids peroxidation, similar to the effect obtained after treatment with anti-Fas antibody and TNFα plus cycloheximide. Consistent with our previous results, tBid required its H6 domain to induce mitochondrial lipid peroxidation. Mitochondrial lipid peroxidation was partially inhibibited by pre-treating the hepatocytes with the antioxidants, Trolox and MnTBAP [Mn(III)tetrakis(4-benzoic acid) porphyrin chloride], and completely inhibited using the mitochondrion-specific antioxidant, mitoQ10 (**Figure 7E**).

Thus, tBid relies on its αH6 helix, but not on its BH3 domain, to inducte superoxide anion production and mitochondrial lipid peroxidation.

## Discussion

We have previously shown that tBid disrupts mitochondrial bioenergetics independently of its BH3 domain [13]. Here, we identify the helix αH6 as the domain responsible for targeting tBid to mitochondrial CL and disrupting mitochondrial bioenergetics.

We show here for the first time, that the helix αH6 interacts with mitochondria through electrostatic interactions involving the lysines 157 and 158 and recapitulates the effect of tBid on mitochondrial bioenergetics. Binding of αH6 leads to an inhibition of ADP-stimulated respiration (state 3 respiration) associated with a slight uncoupling of state-4 respiration. This is consistent with the findings by Liu *et al*. [15] that showed the ability of the Bid's “putative cardiolipin-binding domain”, containing αH6, to affect mitochondrial respiration. Biophysical analyses revealed that, like tBid [13], αH6 binds specifically to CL-enriched lipid monolayers via electrostatic interactions and reorganized them into microdomains. These results, together with our previous report [13], strongly indicate that tBid interacts with mitochondrial contact sites through electrostatic interaction involving its helix αH6 with CL. In parallel, we also demonstrated that tBid required its helix αH6 to induce the production of mitochondrial ROS and to promote mitochondrial lipid peroxidation. Thus, interaction of tBid with CL may lead to the reorganization of the mitochondrial phospholipids into microdomains, and affect the activity the enzymes involved in mitochondrial functions resulting in ROS production and mitochondrial lipid peroxidation.

Moreover, electrostatic interaction of αH6 with mitochondria leads to the release of cytochrome *c* and subsequent caspase activation and apoptosis. Of note, mutation of the lysines 157 and 158 did not affect the mitochondrial localization of tBid but inhibited tBid-cytochrome *c* release activity. This strongly supports a role of αH6 in this process. In line with this, we showed that tBid relies on its αH6 to induce apoptosis.

Importantly, while tBid does not rely on its BH3 domain to translocate to mitochondria and to inhibit mitochondrial function, the BH3 domain is required for the ability of tBid to efficiently induce cytochrome *c* release and apoptosis. This is in aggrement with previous studies showing that tBid interacts with mitochondria in Bax and Bak independent manner [17], [27], [28] and rules out the possibility these two proteins act as putative receptors for tBid at the mitochondrial surface. Altogether, these results indicate that tBid induces apoptosis via a BH3-dependant mechanism involving Bax and Bak and via a BH3-independant mechanism involving electrostatic interactions within its αH6.

**Figure 8:**
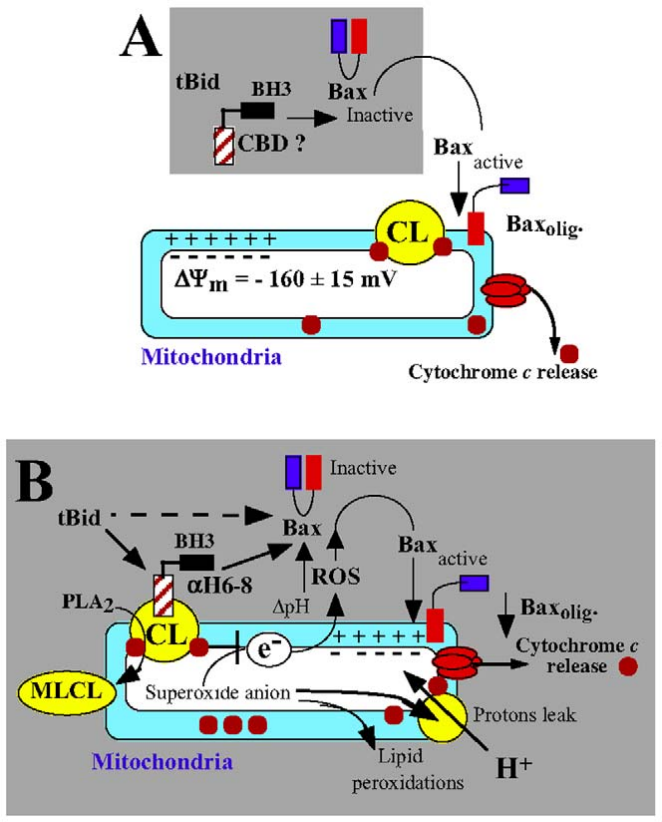
tBid, a bifunctional molecule. (A) Current model of the BH3-dependant function of tBid. tBid interacts through its BH3 domain and directly activates Bax, which undergoes conformational changes that induce the exposure of its N-terminal domains. This results in the stable insertion and subsequent oligomerization of Bax in the mitochondrial outer membrane leading to the release of cytochrome *c* and apoptosis. This model highlights the importance of protein-protein interactions between tBid and Bax. (B) Refined model of the pro-apoptotic function of tBid: importance of tBid/CL interactions. First, tBids binds to CL present at the contact sites via its helix αH6 and destabilizes the mitochondrial membrane. This may affect the activity of the electron transport chain complexes and lead to an acidification of the cytosol, mitochondrial ROS production and mitochondrial lipid peroxidation. This environment may prime the activation of Bax and/or Bak. Then, tBid interacts through its BH3 domain with Bax and/or Bak to promote their oligomerization and subsequently induce cytochrome c release and apoptosis.

Based on these observations, we proposed a two steps model for the pro-apoptotic activity of tBid (**Figure 8**). First, tBid binds to CL present at the mitochondrial contact sites via its helix αH6 and destabilizes mitochondrial functions, resulting in cytosolic acidification, ROS production and mitochondrial lipid peroxidation. This environment may prime the activation of Bax and/or Bak. Notably, hydrogen peroxide-mediated acidification of the cytosol has been shown to promote the translocation of Bax to the mitochondria [29]. Then, tBid interacts through its BH3 domain with Bax/Bak to promote their oligomerization and subsequently induce cytochrome *c* release and apoptosis. We believe that our results provide new insights into the mechanism of action of tBid on the mitochondria and strongly support the importance of protein-lipid interaction, and in particular of the helix αH6 with CL, in this process.

## Materials and Methods

### Materials

All chemicals and supplies were obtained from Sigma (St. Louis, MO, USA) unless otherwise stated. Full-length His-tagged wild-type and mutant mouse Bid were purified as described by Desagher *et al.* [30].

Stock solutions of tBid (1.9 mg/ml) were diluted to nM concentrations in respiratory buffer. Buffer alone had no effect on mitochondrial parameters.

### Animals

We used 6- to 12-week-old, age- and sex-matched congenic mice, expressing a Bcl-2 [31] or Bcl-xL transgene [32] under the L-type pyruvate kinase gene promoter. Bax^−/−^ and Bak^−/−^ mice (kindly provided by Craig.B. Thompson and Stanley Korsmeyer, USA) were also used. C57/Bl6 mice were used as controls and for hepatocytes or mitochondrial preparations.

### Peptides Synthesis

Peptides were synthesized by Covalab (Lyon, France) on a Rainin Symphony synthesizer by a standard SPPS (solid phase peptide synthesis) method (Novabiochem). The peptides were purified by HPLC, and the identity and purity of the peptides were confirmed by mass spectroscopy and by amino acid analysis.

The Bid-BH3 peptide was synthesized with the sequence RNIARHLAQVGDSMDR. Nontoxic concentrations (less than 5 µM) of this peptide were added to mitochondria from Bax/Bak DKO cells. The sequence of the principal helix, αH6, was EKTMLVLALLLAKKVASH; that of its mutated counterpart, αH6m (αH6 modified), was EKTMLVLALLLAAAVASH. The toxicity of the Bid-BH3 peptide and others was tested on purified mitochondria, using the same protocol as for tBid (see below). A Bid-BH3 peptide linked by five glycine residues to H6 was also constructed (EKTMLVLALLLAKKVASH-GGGGGRNIA RHLAQVGDSMDR) and purified to test the efficiency of the BH3 peptide linked to an effector involved in the disruption of mitochondria, namely αH6. All peptides synthetized have an helicoidal alpha structure when membranes containing CL are present. Number of amino acids (AA), isolectric point (pI), hydrophobicity and charge were calculated for tBid and the αH6 and αH6m peptides (Table 1).

The calculation of hydrophobicity was performed using the PROTSCALE software from the Swiss Institute of Bioinformatics (http://www.expasy.org/tools/protscale.html) according to the method of Kyte & Doolittle [33]. All tBid constructs used in this study are described in Figure 5A. They are named tBid, tBidDBH3, tBidDH3–5, tBidDH6, tBidDH6–7, tBidDH6–8, tBidDBH3DH6, tBidDBH3DH6–7, tBid_KK157,158AA_, tBid_E94G_ and also tBid_E94G,KK157,158AA_ (all the constructs are described in Figure 1C exept tBidDH3, tBidDH4 and tBidDH5). Mef Bax^−/−^, Bak^−/−^ and DKO (Bax^−/−^, Bak^−/−^) cells were kindly provided by Stanley Korsmeyer, Dana Farber Institute, Boston, MA, USA.

### DNA Constructs

Full-length Bid cDNA was obtained from HL-60 cells by RT-PCR and subcloned into pCR2.1 vector from Stratagene, Inc (La Jolla, CA, USA). Oligonucleotide primers (Operon Technologies, Alameda, CA, USA) were designed to amplify the C-terminus of Bid (tBid) and deletion mutants by PCR with Bid cDNA as the starting template. Deletion mutants were based on the 3D structure in solution [19], [20]. These PCR products were then subcloned into pGEX-4T-1 (Qiagen, Inc, Alameda,Ca, USA), a GST-fusion vector.

### Transfections

The tBid-EYFP deletion mutants were transiently introduced into AtT20 or CV-1 cells with Lipofectamine 2000™ (Invitrogen SARL, Cergy Pontoise, France). Cells were seeded one day before transfection. We used 2 µg of plasmid DNA and 10 µl Lipofectamine 2000™ for each 6-well plate. Transfected tBid protein induces a high rate of cell death; thus experiments were carried out in the presence of 10 µM Bok-D, a very potent caspase-3 inhibitor, to avoid complete breakdown of the cells following binding of the protein to the mitochondrial membrane and cells were shorted on a low level of EYFP before flow cytometry analysis since higher level linked to overexpression of the contructs led to a cytotoxic death.

### Isolation of Mouse Hepatocytes

Mouse hepatocytes were isolated using a standard protocol [34]. Viable purified hepatocytes were separated from other cells by iso-density Percoll centrifugation [35] and were resuspended in M199 medium (Gibco-BRL) for immediate use.

### Oxygen Uptake, Membrane Potential, Rate of Phosphorylation and Large Amplitude Swelling

Percoll-purified mitochondria from mouse liver [36] were resuspended in respiratory medium (R) consisting of 100 mM KCl, 40 mM Sucrose, 5 mM MgCl_2_, 10 mM TES, 10 mM KH_2_PO_4_ (pH 7.2), 1 mM EGTA and 0.1% BSA. Mitochondrial protein concentration was 0.333 mg/ml, for all procedures (in a 3 ml cuvette). Mitochondrial respiration rate (Vox) and membrane potential (ΔΨm) were monitored by simultaneous use of a Clark-type electrode, a tetraphenylphosphonium cation-sensitive electrode (TPP^+^) and a pH-electrode at 25°C in medium R as described in Gonzalvez *et al*. [13]. Trace amounts of EGTA (10 µM) ensured good experimental reproducibility without affecting pore opening. When mitochondria are extracted from cells, the procedure used is that of Gonzalvez *et al.* [13] and the mitochondria are resuspended in the medium R as descripbed above.

### In-Vitro Assay of Cytochrome c Release

Purified mitochondria from mouse livers were resuspended in medium R (pH 7.2). Isolated mitochondria (5 µL; stock concentration 25 mg/mL) in medium S were gently stirred for 30 min at room temperature with the indicated amount of proteins or reagents in the presence of protease inhibitor cocktail (4 µl) (Sigma) in a final volume of 50 µL. Mitochondria were then collected by centrifugation at 12,000 *g* for 5 min at 4°C; supernatants were run on a 15% SDS-PAGE and cytochrome *c* detected with a mouse monoclonal anti-cytochrome *c* antibody (Pharmingen, clone 7H8.2C12). In other circonstancies, we used the ELISA test from.

### Biophysical Measurement of Lipid Monolayers

We used the purest quality of DPPC (Dipalmitoylphosphatidylcholine) available from Sigma. The sodium salt of bovine heart cardiolipin (BHCL) was purchased from Sigma and used without further purification (fatty acid analysis by gas-liquid chromatography: 18∶2, 92.2%; 18∶1, 6.7%; 16∶0, 0.5%; 16∶1, 0.4%; 18∶0, 0.2%). Synthetic TMCL (Tetramyristoyl cardiolipin) (and fluorescent probes NBD-PC (2-(6-(7-nitrobenz-2-oxa-1, 3-diazol-4-yl) amino) hexanoyl -1-hexadecanoyl-*sn*-glycero-3-phosphocholine) and NBD-PG (2-(6-(7-nitrobenz-2-oxa-1,3-diazol-4-yl)amino) hexanoyl-1-hexadecanoyl-*sn*-glycero-3-phosphoglycerol) were obtained from Molecular Probes Inc. (USA.). Stock solutions (1×10^−3^ M and 2.5×10^−4^ M) were prepared for DPPC in chloroform and for TMCL or BHCL in 3∶1 chloroform/methanol (v/v). Penetration experiments were carried out in a Teflon dish at 22°C in a thermostatically controlled box. The Teflon dish has a volume of 10 ml and a surface area of 12.57 cm^2^. Phospholipid monolayers were spread to give the desired initial surface pressure on a subphase of 150 mM NaCl, 5 mM Tris-HCl (pH 7.5). The subphase was continuously stirred with a magnetic bar at a slow rate so as not to disturb the monolayer. The lipid monolayers then equilibrated for 15 min at various initial surface pressures (π_i_) before injection of the helix into the subphase. Peptide was added to the subphase through a hole in the Teflon chamber. All injected volumes were ≤1% of the total subphase volume. The surface pressure (π) was measured by the Wilhelmy method. Changes in π were completed 30 min after peptide injection. The maximum increase in surface pressure (Δπ) induced by the adsorption of the peptide was defined as the difference between the initial pressure (π_i_) and the plateau value. The data shown are means from triplicate measurements and are represented as Δπ versus π_i_.

### Epifluorescence Microscopy of Lipid Monolayers

Monolayers were visualized with an Olympus-BX30 microscope set on the Teflon dish. As previously described [37], images were recorded with an AIS (MXRi2) video camera. These experiments were carried out at 22°C and pH 5.7. Mixtures were prepared from a chloroform solution of DPPC or TMCL containing 0.5 mol% NBD-PC or NBD-PG. The probe is excluded from densely packed areas of lipid, providing contrast between lipid phases. Images were recorded at constant area and as a function of time after the injection of the peptide.

### Intracellular Localization of tBid and tBid Constructs: Acquisition and Image Analysis

Transfected cells (CV-1) were stained with 20 nM TMRE (tetramethylrhodamine ethyl ester, Molecular Probes, Invitrogen) for 15 min at room temperature. Images were collected in real time on a Nikon Eclipse TE 300 DV inverted microscope fitted with an 100X oil objective using appropriate fluorescence emission filters. Images of EYFP and TMRE (tetramethylrhodamine ethyl ester) fluorescence were acquired with a back-illuminated cooled detector (CCD EEV: NTE/CCD-1024-EB, Roper Scientific, France). NAD(P)H fluorescence was also recorded in order to have a dual-localization reference; NAD(P)H fluorescence is predominantly mitochondrial (80%), but is partly (20%) cytoplasmic. NAD(P)H auto-fluorescence was monitored under excitation wavelengths of 340 to 380 nm and emission wavelengths of 435 to 485 nm. Metamorph software (Universal Imaging Corporation, Roper Scientific, France) was used for data acquisition. The Image J program (http://rsb.info.nih.gov/ij/) was used for image processing.

### Microspectrofluorometric Analysis

A Zeiss UMSP 80 confocal microscope equipped with an UV-visible argon laser and an optical multichannel analyzer (OMA III with an IRY 1024/6B from Princeton Applied Research) was used for microspectrofluorometric analysis under epi-illumination conditions. A Prism system (Jobin & Yvon) was used to acquire and treat data. The excitation wavelength was set to 488 nm. The spectra were recorded at wavelengths in the 540 nm to 750 nm range in an 0.8 mm^2^ area. Spectra for cytoplasmic EYFP (empty vector from BD Bioscience, USA) and mitochondrial TMRE were used as references (Figure 5B).

### Electropermeabilization

The J. Jhan human lymphoblastoid CD4^+^ T-cell line (derived from the Jurkat cell line; provided by J.D. Fox, London, England) was grown at a density of 1×10^6^ cells per milliliter in RPMI 1640 medium supplemented with 5% (v/v) fetal calf serum, 2 mM L-glutamine, 100 U/ml penicillin, and 100 µg/ml streptomycin in 5% CO_2_ at 37°C. All procedures were as described by Gabriel *et al.* [26].

### Western Blot Analysis

Cells were lysed by incubation with modified Laemmli buffer (60 mM Tris, pH 6.8, 10% glycerol and 2% sodium dodecyl sulfate (SDS), without β-mercaptoethanol and bromophenol blue) and sonicated for 30 s on ice. The lysate was centrifuged at 3000 *g* for 5 min. The supernatant was incubated for 5 min at 100°C. Aliquots of the supernatant were assayed for protein concentration (Micro-BCA Protein Assay, Pierce Chemical Co., Rockford, IL, USA). Cell lysate proteins (20 µg per lane) were resolved by 7.5% and 15% SDS-polyacrylamide gel electrophoresis. Proteins were then electroblotted onto nitrocellulose filters with 0.45 µm pores; filters were incubated for 1 h with 5% nonfat milk and 0.1% Tween 20-containing PBS. The filters were then incubated for 1 h at room temperature with 1 µg/ml monoclonal anti-caspase-3 rabbit antiserum (Pharmingen, San Diego, CA, USA) or 1 µg/ml PARP mAb C2.10 (purchased from Dr. Poirier, Quebec, Canada). Blots were washed three times for 10 min with 0.2% Tween 20-containing PBS and incubated for 1 h with peroxidase-labeled anti-mouse or anti-rabbit immunoglobulin (1/5000). Blots were developed using an enhanced chemiluminescence detection system (ECL, Amersham Corp., Cardiff, UK).

### Flow Cytometry

Changes in mitochondrial membrane potential difference (ΔΨm) were evaluated by incubating cells (5×10^5^/ml) for 15 min at 37°C, with DiOC_6_(3) (stock solution 1 mM in ethanol, final concentration 2.5 nM). Cells were then analyzed using the FACScalibur 4 colors cytometer. The fluorescence was collected for DiOC_6_(3), after suitable compensation, in FL-1 channel (530±30 nm) NAD(P)H autofluorescence was elicited with an argon laser (488 nm at 100 mW) and multiline ultraviolet light at 400 mW. Changes in the auto-fluorescence of normal and apoptotic cells were recorded. The light emitted was collected with a 424±40 nm bandpass for the NAD(P)H fluorescence [38]. The pH within single cells was monitored with the fluorescent pH indicator 5-(and-6-)-carboxy-seminaphtho-rhodafluor-1 acetoxy methyl ester acetate (SNARF-1 AM) as described by Petit *et al*. [38].

Caspase-3-like activity was assayed with the cell-permeable fluorogenic caspase substrate PhiPhilux G1D2 (OncoImmunin Inc., Kensington, MD, USA), which contains the GDEVDG sequence. Briefly, native and electropermeabilized cells were harvested and washed twice in PBS buffer. Cells were resuspended in 50 µl of PhiPhilux-containing solution and incubated for 1 h at 37°C in the dark. Then, they were washed and suspended in PBS buffer and analyzed. Exposed phosphatidylserine (PS) on the plasma membrane outer leaflet was measured by staining cells with annexinV-FITC (1 µg/ml) for 10 min at 4°C. AnnexinV-FITC absorption onto the cell surface was monitored by flow cytometry (FL-1); membrane integrity was assayed with propidium iodide (1 µg/ml), which emitted signal in the FL-3 channel (long pass>670 nm), using standard protocols. A minimum of 5×10^3^ events were acquired in list mode and analyzed with Cellquest software (Becton Dickinson).

When the cells are tranfected with tBid vectors, the cells with low EYFP fluorescence where shorted prior their examination, to avoid cells with high levels of EYFP in which the high surexpression of the protein become cytotoxic, regardless of their physiological properties.

### Superoxide Anion and Hydroperoxide Production and Lipid Peroxidation

Briefly, treated cells were incubated with 2.5 µM dihydroethidium for 15 min at 37°C for superoxide anion production. Cells were washed and resuspended in phosphate-buffered saline for flow cytometry. They were then counterstained with 2 µM TOPRO-3 (1 mg/ml stock solution) to monitor cell viability. ROS generated by isolated mitochondria were monitored using the Amplex Red assay for H_2_O_2_ (Molecular Probes, Eugene, OR, USA) [39]. Mitochondria (50 µg protein) were incubated in standard incubation medium supplemented with 2 µM Amplex Red, 1 U/mL horseradish peroxidase, and 0.1 mM EGTA in a 400 µL cuvette at 37 C with continuous stirring. Fluorescence from resorufin, a product of the Amplex Red reaction with H_2_O_2_, was measured in a Perkin-Elmer LS 55 luminescence spectrometer using excitation/emission wavelengths of 550 and 590 nm, respectively. Results were expressed as a percentage of H_2_O_2_ production with succinate + antimycin A (100%). The generation of ROS in TNFα, Fas-antibody- or tBid-mediated effects - in particular, lipid peroxidation - was determined by measuring malondialdehyde (MDA) formation, a common end product of lipid peroxidation, with the Lipid Peroxidation Assay Kit (Oxford Biomedical Research, Oxford, MI). Materials were prepared according to the manufacturer's instructions. MDA standards provided in the kit were used to quantify the amount of MDA formed.

### Experimentation on Vertebrate Animals

* The research involving animals must have been conducted according to the Directive of 24 November 1986 on the approximation of laws, regulations and administrative provisions of the member states regarding the protection of animals used for experimental and other scientific purposes (86/609/EEC).

* All research involving animals has been approved by ethics council of the National Center for Scientific Research (CNRS).

## References

[1] Cory S, Adams JM (2002). The Bcl2 family: regulators of the cellular life-or-death switch.. Nat Rev Cancer.

[2] Petros AM, Olejniczak ET, Fesik SW (2004). Structural biology of the Bcl-2 family of proteins.. Biochim Biophys Acta.

[3] Muchmore SW, Sattler M, Liang H, Meadows RP, Harlan JE (1996). X-ray and NMR structure of human Bcl-xL, and inhibitor of programmed cell death.. Nature.

[4] Adams JM, Cory S (1998). The Bcl-2 protein family: arbiters of cell survival.. Science.

[5] Letai A, Bassik M, Walensky L, Sorcinelli M, Weiler S (2002). Distinct BH3 domains either sensitize or activate mitochondrial apoptosis, serving as prototype cancer therapeutics.. Cancer Cell.

[6] Chen L, Willis SN, Wei A, Smith BJ, Fletcher JI (2005). Differential targeting of prosurvival Bcl-2 proteins by their BH3-only ligands allows complementary apoptotic function.. Mol Cell.

[7] Eskes R, Desagher S, Antonsson B, Martinou JC (2000). Bid induces the oligomerization and insertion of Bax into the outer mitochondrial membrane.. Mol Cell Biol.

[8] Wei MC, Lindsten T, Mootha VK, Weiler S, Gross A (2000). tBID, a membrane-targeted death ligand, oligomerizes BAK to release cytochrome c.. Genes Dev.

[9] Yin XM, Wang K, Gross A, Zhao Y, Zinkel S (1999). Bid-deficient mice are resistant to Fas-induced hepatocellular apoptosis.. Nature.

[10] Li H, Zhu H, Xu CJ, Yuan J (1998). Cleavage of BID by caspase 8 mediates the mitochondrial damage in the Fas pathway of apoptosis.. Cell.

[11] Luo X, Budihardjo I, Zou H, Slaughter C, Wang X (1998). Bid, a bcl-2 interacting protein, mediates cytochrome c release from mitochondria in response to activation of cell surface receptors.. Cell.

[12] Lutter M, Fang M, Luo X, Nishijima M, Xie X (2000). Cardiolipin provides specificity for targeting of tBid to mitochondria.. Nat Cell Biol.

[13] Gonzalvez F, Pariselli F, Dupaigne P, Budihardjo I, Lutter M (2005). tBid interaction with cardiolipin primarily orchestrates mitochondrial dysfunctions and subsequently activates Bax and Bak.. Cell Death Differ.

[14] Scorrano L, Ashiya M, Buttle K, Weiler S, Oakes SA (2002). A distinct pathway remodels mitochondrial cristae and mobilizes cytochrome c during apoptosis.. Dev Cell.

[15] Liu J, Weiss A, Durrant D, Chi NW, Lee RM (2004). The cardiolipin-binding domain of Bid affects mitochondrial respiration and enhances cytochrome c release.. Apoptosis.

[16] Kim TH, Zhao Y, Ding WX, Shin JN, He X (2004). Bid-cardiolipin interaction at mitochondrial contact site contributes to mitochondrial cristae reorganization and cytochrome c release.. Mol Biol Cell.

[17] Gonzalvez F, Schug ZT, Houtkooper RH, Mackenzie ED, Brooks DG (2008). Cardiolipin provides an essential activating platform for caspase-8 on mitochondria.. J Cell Biol.

[18] Nguyen M, Millar DG, Yong VW, Korsmeyer SJ, Shore GC (1993). Targeting of Bcl-2 to the mitochondrial outer membrane by a COOH-treminal signal anchor sequence.. J Biol Chem.

[19] Chou JJ, Li H, Salvesen GS, Yuan J, Wagner G (1999). Solution structure of BID, an intracellular amplifier of apoptotic signaling.. Cell.

[20] McDonnell JM, Fushman D, Milliman CL, Korsmeyer SJ, Cowburn D (1999). Solution structure of the proapoptotic molecule BID: a structural basis for apoptotic agonists and antagonists.. Cell.

[21] Zha J, Weiler S, Oh KJ, Wei MC, Korsmeyer SJ (2000). Posttranslational N-myristoylation of BID as a molecular switch for targeting mitochondria and apoptosis.. Science.

[22] Hu X, Han Z, Wyche JH, Hendrickson EA (2003). Helix 6 of tBid is necessary but not sufficient for mitochondrial binding activity.. Apoptosis.

[23] Garcia-Saez AJ, Mingarro I, Perez-Paya E, Salgado J (2004). Membrane-insertion fragments of Bcl-xL, Bax, and Bid.. Biochemistry.

[24] Oh KJ, Barbuto S, Meyer N, Kim RS, Collier RJ (2004). Conformational changes in BID, a pro-apoptotic BCL-2 family member, upon membrane-binding: A site-directed spin labeling study.. J Biol Chem.

[25] Degli Esposti M (2002). Sequence and functional similarities between pro-apoptotic Bid and plant lipid transfer proteins.. Biochim Biophys Acta.

[26] Gabriel B, Sureau F, Casselyn M, Teissie J, Petit PX (2003). Retroactive pathway involving mitochondria in electroloaded cytochrome c-induced apoptosis. Protective properties of Bcl-2 and Bcl-XL.. Exp Cell Res.

[27] Lindsten T, Ross AJ, King A, Zong WX, Rathmell JC (2000). The combined functions of proapoptotic Bcl-2 family members bak and bax are essential for normal development of multiple tissues.. Mol Cell.

[28] Wei MC, Zong WX, Cheng EH, Lindsten T, Panoutsakopoulou V (2001). Proapoptotic BAX and BAK: a requisite gateway to mitochondrial dysfunction and death.. Science.

[29] Ahmad KA, Iskandar KB, Hirpara JL, Clement MV, Pervaiz S (2004). Hydrogen peroxide-mediated cytosolic acidification is a signal for mitochondrial translocation of Bax during drug-induced apoptosis of tumor cells.. Cancer Res.

[30] Desagher S, Osen-Sand A, Nichols A, Eskes R, Montessuit S (1999). Bid-induced conformational change of Bax is responsible for mitochondrial cytochrome c release during apoptosis.. J Cell Biol.

[31] Lacronique V, Mignon A, Fabre M, Viollet B, Rouquet N (1996). Bcl-2 protects from lethal hepatic apoptosis induced by an anti-Fas antibody in mice.. Nature Med.

[32] de la Coste A, Fabre M, McDonnel N, Porteu A, Gilgenkrantz H (1998). Bcl-XL and Bcl-2 differentially block fas/CD95- and TNFa-induced apoptotic liver injury in transgenic mice.. Am J physiol.

[33] Kyte J, Doolittle RF (1982). A simple method for displaying the hydropathic character of a protein.. J Mol Biol.

[34] Berry MN, Friend DS (1969). High-yield preparation of isolated rat liver parenchymal cells: a biochemical and fine structural study.. J Cell Biol.

[35] Kreamer BL, Staecker JL, Sawada N, Sattler GL, Hsia MT (1986). Use of a low-speed, iso-density percoll centrifugation method to increase the viability of isolated rat hepatocyte preparations.. In Vitro Cell Dev Biol.

[36] Petit PX, O'Connor JE, Grunwald D, Brown SC (1990). Analysis of the membrane potential of rat- and mouse-liver mitochondria by flow cytometry and possible applications.. Eur J Biochem.

[37] Etienne F, Roche Y, Peretti P, Bernard S (2008). Cardiolipin packing ability studied by grazing incidence X-ray diffraction.. Chem Phys Lipids.

[38] Gendron MC, Schrantz N, Métivier D, Kroemer G, Maciorowska Z (2001). Oxidation of pyrimidine nucleotides during Fas- and ceramide-induced apoptosis: correlation with changes in mitochondria, gluthation depletion, intracellular acidification and caspase-3 activation.. Biochem J.

[39] Votyakova TV, Reynolds IJ (2001). DeltaPsi(m)-Dependent and -independent production of reactive oxygen species by rat brain mitochondria.. J Neurochem.

